# A Verification of Camphor

**Published:** 1885-12

**Authors:** 


### A VERIFICATION OF CAMPHOR.

Planet (Saint Pons) publishes the following case in the *Brussels Med. Psycholog.* of March, 1885: A young man, otherwise healthy, but somewhat nervous and inclined to be his own physician for slight ailments, took for sleeplessness, while suffering from a coryza, about twenty grains of Camphor in small pieces. He soon felt chilly, and a state of something similar to unconsciousness set in, and when he recovered he felt as if crazy. This repeated itself several times, till an emetic removed a large quantity of Camphor. He still felt a sensation of coldness from the stomach down the lower extremities, the hands appeared to him paralyzed, and all that he looked at trembled around him. After a few days' rest he returned to his work, but after three weeks, lateral headaches, sensation of globus, loss of memory, becoming easily frightened, palpitations, hallucinations, alternate weeping and laughing, pollutions without sexual ideas, and a state of somnambulism set in, and some time passed before he felt well again.

Allen, II, 424, gives us similar mental symptoms and many illusions of vision; 384, pain in the epigastrium, radiating all over the belly and into the limbs, coldness only in stomach; 414, cold sensation in upper and lower portion of abdomen; 484, incomplete erection, with weak venereal desire, which soon again vanished; emissions for several nights; nocturnal pollution without dreams; 540, palpitations; 607, limbs are difficult to move; 704, relaxation and heaviness of the whole body; 764, symptoms of intoxication, optical delusions, fright, screams, hideous sights; 816, chilliness over the whole body, etc.

As Cholera and Camphor go hand in hand, it may be worth while to lead our attention to the persistent action of large doses of Camphor, so that we may keep in mind our devise, *Die milde macht ist gross.*

